# Hyperbolic Spatial Covariance Modeling with Adaptive Signal Filtering for Robust Wi-Fi Indoor Positioning

**DOI:** 10.3390/s25134125

**Published:** 2025-07-02

**Authors:** Wenxu Wang, Mingxiang Liu

**Affiliations:** 1 School of Information Engineering, Shaoguan University, Shaoguan 512158, China; wangwenxu0909@sgu.edu.cn; 2 School of Automation and Intelligent Manufacturing, Southern University of Science and Technology, Shenzhen 518055, China

**Keywords:** indoor positioning, spatial covariance modeling, hyperbolic manifold, channel state information, graph attention networks, adaptive filtering

## Abstract

Robust indoor positioning, crucial to modern location-based services, increasingly leverages Channel State Information (CSI) for its superior multipath resolution over the traditional RSSI. However, current CSI-based methods are hampered by three key limitations: susceptibility to skewed, non-Gaussian noise; informational redundancy from multi-AP configurations; and spatial discontinuities arising from Euclidean-based modeling. To address these challenges, we propose a unified framework that synergistically combines three innovations: (1) an adaptive filtering pipeline that uses wavelet decomposition and dynamic Kalman updates to suppress skewed noise; (2) a graph attention network that optimizes AP selection by modeling spatiotemporal correlations; and (3) a hyperbolic covariance model that captures the intrinsic non-Euclidean geometry of signal propagation. Evaluations on experimental data demonstrate that our framework achieves superior positioning accuracy and environmental robustness over state-of-the-art methods. Crucially, the hyperbolic representation enhances resilience to obstructions by preserving the signal manifold’s true structure, thereby advancing the practical deployment of fingerprinting systems.

## 1. Introduction

The proliferation of smart mobile devices has catalyzed revolutionary advancements in location-based services (LBSs), with precise indoor positioning emerging as a critical enabler for applications ranging from asset tracking to augmented reality. Unlike outdoor environments where Global Navigation Satellite Systems (GNSSs) are effective, indoor localization faces fundamental challenges, including severe multipath propagation, Non-Line-of-Sight (NLOS) conditions, and dynamic environmental interference [1]. Among competing technologies, Wi-Fi infrastructure demonstrates distinct advantages. Bluetooth-based systems [2] encounter limitations in coverage density and synchronization complexity, and ZigBee networks [3] sacrifice data rates for energy efficiency, while RFID [4] and UWB [5] require specialized hardware incompatible with existing infrastructure. Leveraging existing Wi-Fi access points (APs), therefore, provides a cost-effective and scalable foundation for indoor positioning, making it a highly viable solution for widespread deployment without additional specialized hardware [6].

Within Wi-Fi-based positioning, early systems predominantly relied on the Received Signal Strength Indicator (RSSI) as the primary signal feature [7]. The RSSI provides a single scalar value representing the total power of the received signal. However, this simplicity is also its fundamental weakness. As a coarse-grained metric, the RSSI is the superposition of signals from all propagation paths, which suffers from severe temporal fluctuations and spatial ambiguity in complex indoor environments, fundamentally constraining the achievable positioning accuracy [8].

To overcome the inherent limitations of the RSSI, recent advancements have shifted the focus to Channel State Information (CSI), a fine-grained physical layer metric [9]. The superiority of CSI over the RSSI for indoor positioning is threefold [1]. First, CSI offers superior multipath resolution; unlike the RSSI, which aggregates all signal paths into one value, CSI provides frequency-selective measurements across dozens of subcarriers, enabling the discrimination of distinct multipath components. Second, CSI provides richer information; it is a high-dimensional vector containing both amplitude and phase information for each subcarrier, offering a detailed channel fingerprint that is far more descriptive than a single power value. Third, the overall structure of the Channel Frequency Response (CFR) that CSI represents exhibits significantly higher temporal stability compared with the volatile RSSI, making it a more robust feature for creating reliable location fingerprints.

Despite its advantages, leveraging CSI for robust positioning introduces its own set of challenges, which remain inadequately addressed by the existing literature. Current methods exhibit three primary limitations. First, raw CSI data are often corrupted by non-Gaussian, skewed noise induced by dynamic environmental factors, which conventional filters assuming simple noise distributions fail to effectively suppress [10,11]. Second, the high dimensionality of CSI from multiple APs often leads to informational redundancy, and existing feature extraction techniques frequently lack adaptive fusion mechanisms to model the complex spatiotemporal correlations between APs, thereby limiting feature discriminability [12,13]. Third, and most critically, the majority of current positioning algorithms rely on Euclidean distance metrics to model spatial relationships. This assumption of a flat, linear space fundamentally fails to capture the intrinsic non-linear manifold structure of signal propagation in complex indoor environments, leading to significant information loss and degraded performance, especially under NLOS conditions [14,15].

In summary, to address the limitations of conventional fingerprinting-based indoor positioning systems, this paper proposes a comprehensive Wi-Fi fingerprinting framework with three key innovations. The main contributions are as follows:

1. Skewness-Adaptive Signal Filtering: To mitigate non-Gaussian noise characteristics induced by multipath propagation, we develop a multi-modal preprocessing pipeline combining wavelet packet decomposition, dynamic Kalman filtering, and skewness-aware normalization. This approach effectively handles asymmetric signal distributions while preserving transient features through adaptive noise covariance updates, outperforming conventional median/Gaussian filters in computational efficiency.
2. Correlation-Driven AP Selection: We introduce a hierarchical feature extraction mechanism that integrates multi-scale Temporal Convolutional Networks with graph attention fusion. This architecture dynamically selects optimal antenna pairs by modeling spatiotemporal correlations between APs through learned attention weights, resolving redundancy issues in conventional static AP selection methods.
3. Hyperbolic Spatial Covariance Modeling: A novel manifold-aware fingerprinting framework that encodes location relationships in hyperbolic space is proposed. By constructing hybrid covariance kernels combining hyperbolic distance metrics and feature similarity, our method better preserves spatial continuity in complex environments compared with Euclidean-based approaches.

The remainder of this paper is organized as follows: Section 2 systematically reviews foundational concepts of Channel State Information characterization and critically examines existing CSI-based indoor positioning strategies. Section 3 mathematically formalizes the indoor positioning problem while presenting the comprehensive system architecture. Section 4 elaborates on the proposed framework, encompassing three synergistic components: multi-modal signal preprocessing, hierarchical spatiotemporal feature extraction, and hyperbolic manifold-aware optimization. Section 5 provides rigorous quantitative comparisons with state-of-the-art methods, accompanied by error distribution analysis and ablation studies. Finally, Section 6 synthesizes key findings, discusses practical implications, and outlines future research trajectories in non-Euclidean signal modeling and edge computing integration.

## 2. Related Work

### 2.1. Channel State Information

Channel State Information (CSI) fundamentally characterizes the propagation medium between wireless transceivers, encapsulating critical parameters including signal attenuation, multipath scattering effects, and environmental interference patterns. As a time-varying descriptor of channel quality, CSI enables the real-time optimization of transmission parameters through adaptive modulation and beamforming techniques, thereby enhancing both spectral efficiency and link reliability. In frequency-domain analysis, the Channel Frequency Response for the *k*-th subcarrier is mathematically expressed as

(1) $$\begin{array}{c} H\left(f_{k}\right)=∥H(f_{k})∥e^{j∠H}, \end{array}$$

where $∥H(f_{k})∥$ and ∠H denote the amplitude and phase components at subcarrier frequency $f_{k}$, respectively. Modern orthogonal frequency-division multiplexing (OFDM) systems facilitate CSI acquisition through commercial network interfaces, generating complex-valued channel matrices that capture spatial–frequency characteristics across multiple antennas. The superiority of CSI over the Received Signal Strength Indicator (RSSI) manifests in three key aspects: (1) frequency-selective resolution through orthogonal subcarriers; (2) phase coherence information for multipath analysis; and (3) spatial diversity via multiple-input multiple-output (MIMO) configurations. Xiao et al. pioneered CSI-based localization through their Fine-Grained Indoor Fingerprinting System (FIFS) [16], employing averaged amplitude features for position estimation. Subsequent research by Chapre et al. [17] enhanced fingerprint dimensionality through MIMO-OFDM implementations, while Wang et al. [12] introduced deep learning architectures for CSI denoising and feature extraction. Further advancements such as PhaseFi [18] demonstrated the viability of calibrated phase information, while other works explored contrastive learning [19] and hybrid CSI-RSSI systems [20]. However, these foundational works, while establishing the richness of CSI features, often overlooked the inherent spatial continuity of the wireless channel—a critical property where signal characteristics vary smoothly across adjacent physical locations. This oversight limits their ability to model gradual signal variations in continuous space, setting the stage for more advanced spatial modeling techniques.

### 2.2. CSI-Based Indoor Positioning Methods

Indoor positioning leveraging CSI has evolved significantly, with model-enhanced fingerprinting emerging as the dominant paradigm. Unlike geometric methods (e.g., AOA [21] and TOF [22]), which falter under Non-Line-of-Sight (NLOS) conditions [23,24], or traditional fingerprinting [16], which suffers from temporal instability and scalability issues [25,26], deep learning-based models can learn complex, non-linear mappings from CSI to location. However, even recent state-of-the-art methods exhibit critical limitations that motivate our work.

A prominent research trend involves using attention mechanisms to enhance feature robustness. For instance, Wan et al. proposed ACPNet, which incorporates channel and spatial attention to dynamically weigh different subcarriers and antenna streams within a single CSI sample [27]. This allows the model to focus on more informative signal components, improving accuracy. The primary limitation of such approaches, however, is that their attention mechanisms are “inward-facing”—they operate within a single CSI matrix to refine its features. While beneficial, this fails to explicitly model the dynamic spatiotemporal correlations between different access points (APs). This oversight leads to suboptimal feature fusion, as the relative reliability of signals from different APs is not dynamically assessed.

To address the issue of spatial relationships, Graph Neural Networks (GNNs) have been introduced to model the physical topology of the indoor environment. The work by Wang et al. represents a state-of-the-art example, constructing a graph to model the connectivity between reference points and APs, even incorporating an AP selection mechanism to reduce redundancy [28]. While GNNs effectively capture network topology better than methods like DeepFi [12], which treat fingerprints as isolated data, they suffer from a “Euclidean blind spot”. These models implicitly assume that the underlying signal propagation manifold is flat. This assumption breaks down in complex indoor environments where obstacles create a non-Euclidean geometric structure. Using Euclidean distance to measure similarity in these graph models leads to a fundamental misrepresentation of the true “signal distance” between points, particularly degrading performance under NLOS conditions, a challenge also faced by hybrid methods like Gridloc [15].

Another key research direction aims to improve the practicality of fingerprinting by reducing the burden of data collection. The Adapted Mean Teacher (AMT) model proposed by Zhu et al. is a notable example, employing a semi-supervised learning framework to leverage a large number of unlabeled data, thereby reducing the need for laborious manual labeling [29]. While this approach commendably tackles the challenge of data quantity, it does not fully address the issue of data quality. Specifically, such methods often rely on standard data augmentation and do not incorporate sophisticated, physics-aware noise models. They largely fail to account for the highly skewed, non-Gaussian noise distributions that are common in real-world CSI data corrupted by dynamic multipath interference. This gap highlights the need for more advanced signal preprocessing pipelines.

In summary, despite significant progress with methods leveraging contrastive learning [30] and graph networks [31], existing approaches face three critical limitations that our work aims to address. First, they lack robust mechanisms for handling the skewed, non-Gaussian noise prevalent in dynamic indoor environments. Second, their feature fusion strategies are often suboptimal, as they do not dynamically model the spatiotemporal correlations between signals from multiple APs. Third, and most critically, their reliance on Euclidean-based metrics fails to preserve the intrinsic non-Euclidean manifold structure of signal propagation, leading to information loss and reduced accuracy in complex spaces. Our framework overcomes these specific challenges through three synergistic innovations: (1) a skewness-adaptive filtering pipeline for superior noise suppression, (2) a graph attention network for correlation-aware AP feature fusion, and (3) a hyperbolic covariance model that provides a more geometrically faithful representation of the spatial relationships in CSI fingerprints.

## 3. Problem Description

In the process of indoor positioning based on the fingerprinting method, signal features are collected in two main stages: an offline initialization stage (for building the fingerprint database) and an online positioning stage. During the initialization stage, we collect fingerprint information at *I* known reference locations within an environment where one or more wireless access points are deployed. At each location $p_{i}$ (i=1,2,…,I), we capture *M* measurements of Channel State Information (CSI), denoted by $g_{i,m}\in R^{N}$ for m=1,2,…,M. We use $p^{⊤}=\left[{p_{1}}^{⊤},{p_{2}}^{⊤},\ldots ,{p_{I}}^{⊤}\right]$ to denote all the reference locations and ${p_{m}}^{⊤}=\left[{g_{1,m}}^{⊤},{g_{2,m}}^{⊤},\ldots ,{g_{I,m}}^{⊤}\right]$ to denote the *m*-th measurement vector at each location. The mean fingerprint is given by $\bar{g}=\frac{1}{M}\sum_{m=1}^{M}g_{m}$. To provide the requested clarification, each measurement vector $g_{i,m}$ (and similarly the online measurement vector y) is composed of the real-valued amplitudes of the Channel Frequency Response (CFR) across *N* subcarriers, as defined in Equation (1):

(2) $$g_{i,m}={\left[∥H_{i,m}\left(f_{1}\right)∥,∥H_{i,m}\left(f_{2}\right)∥,\ldots ,∥H_{i,m}\left(f_{N}\right)∥\right]}^{⊤}\in R^{N}$$

where $H_{i,m}\left(f_{k}\right)$ denotes the complex channel response at location $p_{i}$ for measurement *m* and subcarrier *k*. The target measurement vector y, captured during the online phase, follows the same structure:

(3) $$y={\left[∥H_{\mathrm{target}}\left(f_{1}\right)∥,∥H_{\mathrm{target}}\left(f_{2}\right)∥,\ldots ,∥H_{\mathrm{target}}\left(f_{N}\right)∥\right]}^{⊤}$$

**Figure 1:**
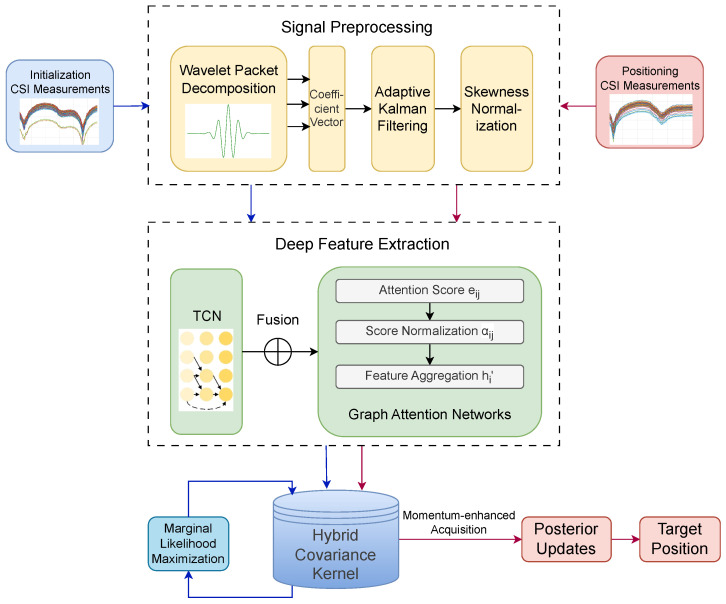
Flowchart of the proposed positioning algorithm. The workflow consists of four sequential stages: signal preprocessing, feature extraction, manifold modeling, and adaptive positioning.

In the positioning stage, our main task is to estimate the receiver’s position coordinates $p_{\mathrm{target}}$ based on the received measurement vector y. The schematic diagram of the two stages of fingerprint positioning is shown in Figure 1.

## 4. Methodology

The proposed indoor positioning methodology consists of an offline phase for database construction and model training and an online phase for estimating the position of a target based on its received signal.

### 4.1. Offline Phase: Database Preparation and Model Training

#### 4.1.1. Fingerprint Database Acquisition

As described in Section 3, an initial database is collected, containing CSI measurements $g_{i,m}\in R^{N}$ for m=1,…,M samples at $N_{fp}$ distinct reference locations $p_{i}$ ($i=1,\ldots ,N_{fp}$). We denote the physical positions by $P=\{p_{1},\ldots ,p_{N_{fp}}\}$. For robustness, the *M* measurements at each location can be averaged or processed to yield a representative signal vector ${\bar{g}}_{i}$ for each location $p_{i}$.

#### 4.1.2. Signal Preprocessing

Each representative signal vector ${\bar{g}}_{i}$ (or potentially each raw $g_{i,m}$) undergoes a three-stage preprocessing pipeline to mitigate noise and artifacts inherent in CSI measurements. Let $s_{raw}$ denote a signal vector input to this stage. The sequential design of this pipeline is motivated by the hierarchical nature of noise in CSI signals and empirical validation across diverse environments. Wavelet decomposition first isolates high-frequency noise components, Kalman filtering then tracks low-frequency signal dynamics, and skewness normalization finally addresses asymmetric distortions from multipath propagation. This order prevents distortion amplification (e.g., normalizing before filtering would amplify Kalman’s residual errors) and aligns with the noise characteristics observed in our experimental data.

##### Wavelet Packet Decomposition

The raw signal $s_{raw}\in R^{N}$ is decomposed using a 3-level wavelet packet decomposition with Daubechies-4 basis functions. The Daubechies-4 (db4) wavelet was specifically chosen as it offers an optimal trade-off between compact support and regularity for analyzing CSI signals. Its support is narrow enough to accurately localize the sharp, high-frequency transients caused by multipath effects, while its four vanishing moments provide sufficient smoothness to model the underlying signal structure without introducing artifacts or blurring critical features. This balance is crucial to effectively separating the stable location fingerprint from volatile noise, outperforming both simpler (e.g., Haar) and more complex (e.g., db8) alternatives for this application. The wavelet packet functions are defined as

(4) $$\psi_{k,n}\left(t\right)=2^{k/2}\psi \left(2^{k}t-n\right),\;k\in \left\{1,2,3\right\},\;n\in Z$$

where ψ(t) is the mother wavelet. This yields $2^{3}=8$ subbands of wavelet coefficients $\left\{d_{k,n}\right\}$.

(5) $$s_{raw}\approx \sum_{k=1}^{8}\sum_{n}d_{k,n}\psi_{k,n}\left(t\right)$$

Optimal subbands might be selected based on criteria like Shannon entropy to retain the most informative components:

(6) $$E\left(d_{k}\right)=-\sum_{n}\frac{|d_{k,n}{|}^{2}}{∥d_{k}{∥}^{2}}\log\frac{|d_{k,n}{|}^{2}}{∥d_{k}{∥}^{2}}$$

##### Adaptive Kalman Filtering

The selected detail coefficients (let $z_{t}$ be the vector of coefficients at index/time *t*) are filtered using a Kalman filter to reduce noise while adapting to signal dynamics. The state $s_{t}$ represents the estimated true coefficients.

(7) $$\begin{array}{ccc} s_{t}^{-} & =s_{t-1}\; & (\mathrm{State}\;\mathrm{prediction}) \end{array}$$

(8) $$\begin{array}{ccc} P_{t}^{-} & =P_{t-1}+Q\; & (\mathrm{Covariance}\;\mathrm{prediction}) \end{array}$$

(9) $$\begin{array}{ccc} K_{t} & =P_{t}^{-}{\left(P_{t}^{-}+R_{t}\right)}^{-1}\; & (\mathrm{Kalman}\;\mathrm{Gain}) \end{array}$$

(10) $$\begin{array}{ccc} s_{t} & =s_{t}^{-}+K_{t}\left(z_{t}-s_{t}^{-}\right)\; & (\mathrm{State}\;\mathrm{update}) \end{array}$$

(11) $$\begin{array}{ccc} P_{t} & =\left(I-K_{t}\right)P_{t}^{-}\; & (\mathrm{Covariance}\;\mathrm{update}) \end{array}$$

Here, $z_{t}$ is the observed coefficient vector at step *t*, Q is the process noise covariance (e.g., 0.01I), and $R_{t}$ is the measurement noise covariance, updated adaptively as

(12) $$R_{t}=\left(1-\alpha \right)R_{t-1}+\alpha \left(z_{t}-s_{t}^{-}\right){\left(z_{t}-s_{t}^{-}\right)}^{⊤}$$

with a forgetting factor α (e.g., 0.1). Let $\hat{s}$ denote the vector of filtered coefficients after processing all relevant indices/times.

##### Robust Skewness Normalization

The filtered coefficients $\hat{s}$ are normalized to handle potential skewness in their distribution by using median and Interquartile Range (IQR), compensated by a skewness factor:

(13) $$\tilde{s}=\frac{\hat{s}-\mathrm{median}\left(\hat{s}\right)}{\mathrm{IQR}(\hat{s})}⊙\tanh(\gamma |\hat{s}\left|\right)$$

where ⊙ denotes element-wise multiplication and γ depends on the sample skewness of $\hat{s}$, which is

(14) $$\mathrm{skewness}\left(\hat{s}\right)=\frac{\frac{1}{N}\sum_{j=1}^{N}{\left({\hat{s}}_{j}-\mu \right)}^{3}}{\sigma^{3}},\;\gamma =c\cdot \left|\mathrm{skewness}\left(\hat{s}\right)\right|$$

with μ,σ being the mean and standard deviation of $\hat{s}$ and *c* being a scaling constant (e.g., 1.5). Let ${\tilde{s}}_{i}$ denote the preprocessed signal corresponding to location $p_{i}$.

#### 4.1.3. Deep Feature Extraction

The preprocessed signals ${\tilde{s}}_{i}$ are fed into a deep learning model to extract discriminative feature vectors $h_{i}$. This model combines temporal and spatial information.

##### Multi-Scale Temporal Encoding

Temporal dependencies are captured using parallel Temporal Convolutional Network (TCN) branches with different dilation factors (e.g., d∈{1,2,4}). Each TCN layer performs a convolution:

(15) $$h_{\mathrm{tcn}}^{\left(l\right)}=\mathrm{ReLU}\left(\sum_{k=0}^{K-1}W_{k}^{\left(l\right)}{\tilde{s}}_{t-dk}+b^{\left(l\right)}\right)$$

Weights $W_{k}^{\left(l\right)}$ are initialized, e.g., using Xavier initialization:

(16) $$W_{k}^{\left(l\right)}∼N\left(0,\sqrt{\frac{2}{n_{\mathrm{in}}+n_{\mathrm{out}}}}\right)$$

The outputs from parallel branches can be concatenated or summed.

##### Graph Attention Fusion

If multiple access points (APs) or receiving antennas are used, spatial relationships are modeled using graph attention networks (GATs). Features from different sources (APs/antennas) corresponding to the same location are fused. Let $\left\{h_{j}\right\}$ be the TCN features from neighboring sources $j\in N_{i}$ (within communication range or physically adjacent). The attention mechanism computes weights $\alpha_{ij}$:

(17) $$\begin{array}{cc} e_{ij} & =\mathrm{LeakyReLU}(a^{⊤}\left[Wh_{i}∥Wh_{j}\right]) \end{array}$$

(18) $$\begin{array}{cc} \alpha_{ij} & =\frac{\exp(e_{ij})}{\sum_{k\in N_{i}}\exp\left(e_{ik}\right)} \end{array}$$

The fused feature $h_{i}^{\prime }$ for source *i* is then

(19) $$h_{i}^{\prime }=\mathrm{ELU}\left(\sum_{j\in N_{i}}\alpha_{ij}Wh_{j}\right)$$

where W and a are learnable parameters of the GAT layer. The final output of this stage is a feature vector $h_{i}\in R^{D}$ for each reference location $p_{i}$. The TCN and GAT models are trained offline using the preprocessed fingerprint data ${\left\{\left(p_{i},{\tilde{s}}_{i}\right)\right\}}_{i=1}^{N_{fp}}$, typically aiming to optimize a loss function related to position estimation or feature discriminability. Let the resulting feature database be $D_{h}={\left\{\left(p_{i},h_{i}\right)\right\}}_{i=1}^{N_{fp}}$.

#### 4.1.4. Manifold-Aware Fingerprint Modeling (GP Training)

We model the relationship between position p and the extracted feature vector h using a Gaussian Process (GP). The GP assumes h=f(p)+ϵ, where f(p) is a function drawn from a GP prior and ϵ is noise. The key component is the covariance kernel function $K(p_{i},p_{j})$, which defines the similarity between features at different locations.

##### Hybrid Covariance Kernel

Conventional fingerprinting methods typically assume that the spatial relationship between reference points can be adequately modeled in Euclidean space. However, this assumption falters in complex indoor environments characterized by significant multipath propagation and Non-Line-of-Sight (NLOS) conditions. The physical obstacles, such as walls and furniture, impose a natural hierarchical structure on the signal propagation paths. For example, signals from a corridor access point to locations in different adjacent rooms must all traverse their respective doorways, creating a tree-like signal path topology where the corridor acts as a parent node. It has been well-established that such hierarchical or tree-like structures are more faithfully represented in negatively curved hyperbolic spaces than in flat Euclidean spaces, as the exponential volume growth of hyperbolic manifolds allows for low-distortion embeddings  [32,33]. The mathematical prerequisite for employing a hyperbolic metric, therefore, arises directly from the intrinsic geometry of the indoor signal manifold. Using hyperbolic distance allows the model to capture the effective “signal distance” between points, which is more relevant for positioning than the direct Line-of-Sight Euclidean distance. We, therefore, propose a hybrid kernel that combines this global geometric prior with local feature-based information:

(20) $$K\left(p_{i},p_{j};\ell ,\eta \right)=\exp\left(-\frac{d_{H}{\left(p_{i},p_{j}\right)}^{2}}{2\ell^{2}}\right)+\eta \frac{h_{i}^{⊤}h_{j}}{∥h_{i}∥∥h_{j}∥}+\sigma_{n}^{2}\delta_{ij}$$

where

- $d_{H}\left(p_{i},p_{j}\right)$ is the hyperbolic distance between positions $p_{i}$ and $p_{j}$. **Note:** The specific formula depends on the chosen hyperbolic model (e.g., Poincare disk/half-plane) and the mapping from Euclidean coordinates p to hyperbolic space, which needs to be defined.
- *ℓ* is the characteristic length scale in the hyperbolic space.
- $h_{i},h_{j}$ are the feature vectors corresponding to $p_{i},p_{j}$. The cosine similarity $\frac{h_{i}^{⊤}h_{j}}{∥h_{i}∥∥h_{j}∥}$ is used for the feature similarity component.
- η weighs the contribution of feature similarity.
- $\sigma_{n}^{2}$ is the noise variance, and $\delta_{ij}$ is the Kronecker delta.

##### Kernel Parameter Optimization

The hyperparameters $\theta =\{\ell ,\eta ,\sigma_{n}^{2}\}$ are learned offline by maximizing the log marginal likelihood of the observed fingerprint features $H={\left[h_{1},\ldots ,h_{N_{fp}}\right]}^{⊤}$ given the positions P:

(21) $$\theta^{*}=\arg\max_{\theta }\log p\left(H|P,\theta \right)$$

Assuming independent features for simplicity or considering a multi-output GP framework, the log marginal likelihood involves the Gram matrix K, whose entries are $K_{ij}=K\left(p_{i},p_{j};\theta \right)$. For a single dimension of the feature vector (let $h^{\left(d\right)}$ be the d-th dimension across all points), this is

(22) $$\log p\left(h^{\left(d\right)}|P,\theta \right)=-\frac{1}{2}{\left(h^{\left(d\right)}\right)}^{⊤}K^{-1}h^{\left(d\right)}-\frac{1}{2}\log\left|K\right|-\frac{N_{fp}}{2}\log\left(2\pi \right)$$

This optimization is typically performed using gradient-based methods.

### 4.2. Online Phase: Target Positioning

A new CSI measurement $y_{\mathrm{target}}$ from an unknown target location $p_{\mathrm{target}}$ is given.

#### 4.2.1. Preprocessing and Feature Extraction

The target signal $y_{\mathrm{target}}$ is processed through the same pipeline used in the offline phase (Section 4.1.2 and Section 4.1.3) to obtain its feature vector $h_{\mathrm{target}}$.

#### 4.2.2. Bayesian Optimization for Position Estimation

We use the trained GP model $h∼\mathrm{GP}(0,K\left(p,p^{\prime };\theta^{*}\right))$ and the target feature vector $h_{\mathrm{target}}$ to estimate the target’s position $p_{\mathrm{target}}$. We aim to find the position p that maximizes the likelihood of observing $h_{\mathrm{target}}$. The log likelihood (for a single feature dimension $h_{\mathrm{target}}^{\left(d\right)}$) is

(23) $$\log p\left(h_{\mathrm{target}}^{\left(d\right)}|p,D_{h},\theta^{*}\right)=-\frac{{\left(h_{\mathrm{target}}^{\left(d\right)}-\mu^{\left(d\right)}\left(p\right)\right)}^{2}}{2\sigma^{2}\left(p\right)}-\frac{1}{2}\log\left(2\pi \sigma^{2}\left(p\right)\right)$$

where $\mu^{\left(d\right)}\left(p\right)$ and $\sigma^{2}\left(p\right)$ are the GP predictive mean and variance for the d-th feature dimension at query location p:

(24) $$\begin{array}{cc} \mu^{\left(d\right)}\left(p\right) & =k{\left(p\right)}^{⊤}{\left(K+\sigma_{n}^{2}I\right)}^{-1}h^{\left(d\right)} \end{array}$$

(25) $$\begin{array}{cc} \sigma^{2}\left(p\right) & =K\left(p,p\right)-k{\left(p\right)}^{⊤}{\left(K+\sigma_{n}^{2}I\right)}^{-1}k\left(p\right) \end{array}$$

with $k\left(p\right)={\left[K\left(p,p_{1}\right),\ldots ,K\left(p,p_{N_{fp}}\right)\right]}^{⊤}$ and K being the Gram matrix computed on the fingerprint locations P. Assuming independence across feature dimensions, the total log likelihood is the sum over dimensions *d*. We use Bayesian Optimization (BO) to maximize this log likelihood function $L\left(p\right)=\sum_{d}\log p\left(h_{\mathrm{target}}^{\left(d\right)}|p,D_{h},\theta^{*}\right)$ over the possible location space p.

##### Momentum-Enhanced Acquisition Function

BO iteratively selects the next point $p_{t+1}$ to evaluate by maximizing an acquisition function. We use Expected Improvement (EI) enhanced with a momentum term:

(26) $$\alpha \left(p\right)=\mathrm{EI}\left(p\right)+\beta m_{t}^{⊤}\nabla_{p}\mathrm{EI}\left(p\right)$$

where $m_{t}$ is a momentum term accumulating past gradients of EI and β controls its influence. The standard EI for maximizing the objective function L(p) is

(27) $$\mathrm{EI}\left(p\right)=\left(\mu_{L}\left(p\right)-L^{+}\right)\Phi \left(Z\right)+\sigma_{L}\left(p\right)\phi \left(Z\right)$$

with $L^{+}$ being the maximum log likelihood value observed so far and

(28) $$Z=\frac{\mu_{L}\left(p\right)-L^{+}}{\sigma_{L}\left(p\right)}$$

Here, $\mu_{L}\left(p\right)$ and $\sigma_{L}\left(p\right)$ are the mean and standard deviation of the GP model built for the log likelihood function L(p) itself. (Note: This requires a secondary GP model within the BO loop or approximations.) Φ(·) and ϕ(·) are the standard normal CDF and PDF.

##### Posterior Updates (Within BO)

After evaluating $L(p_{t})$ at the selected point, the BO’s internal GP model for L(p) is updated. The BO procedure continues for a fixed number of iterations $T_{\max}$ or until convergence. The final estimated position ${\hat{p}}_{\mathrm{target}}$ is the location p that yielded the highest log likelihood L(p) during optimization.

### 4.3. Algorithm Summary

The overall process is summarized in Algorithm 1.
**Algorithm 1** Manifold-aware Bayesian Positioning 1:  **Input:** Initial fingerprint database $D=\{\left(p_{i},g_{i,m}\right)\}$, Target signal $y_{\mathrm{target}}$ 2:  **Output:** Estimated target position ${\hat{p}}_{\mathrm{target}}$  **Offline Phase:** 3:  Preprocess all $g_{i,m}$ (or averages ${\bar{g}}_{i}$) to get ${\tilde{s}}_{i}$ (Equations (5)–(13)) 4:  Train Deep Feature Extractor (TCN/GAT) using $\left\{\left(p_{i},{\tilde{s}}_{i}\right)\right\}$ 5:  Extract features for all reference points: $h_{i}=\mathrm{Extractor}\left({\tilde{s}}_{i}\right)$. Create $D_{h}={\left\{\left(p_{i},h_{i}\right)\right\}}_{i=1}^{N_{fp}}$ 6:  Optimize GP kernel hyperparameters $\theta^{*}=\left\{\ell^{*},\eta^{*},\sigma_{n}^{2*}\right\}$ using $D_{h}$ by maximizing marginal likelihood (Equation (21)) 7:  Construct the final Gram matrix K using $\theta^{*}$ and P  **Online Phase:** 8:  Preprocess target signal $y_{\mathrm{target}}$ to get ${\tilde{s}}_{\mathrm{target}}$ 9:  Extract target feature $h_{\mathrm{target}}=\mathrm{Extractor}\left({\tilde{s}}_{\mathrm{target}}\right)$10:  Define the log-likelihood objective function $L\left(p\right)=\log p\left(h_{\mathrm{target}}|p,D_{h},\theta^{*}\right)$ based on GP predictions (Equations (23)–(25))11:  Initialize Bayesian Optimization to maximize L(p).12:  **for** $t=1\;\mathrm{to}\;T_{\max}\;\mathrm{do}$13:        Select next query point $p_{t}$ by maximizing acquisition function α(p) (Equation (26))14:        Evaluate $L(p_{t})$15:        Update BO’s internal model of L(p)16:  **end for**17:  Return ${\hat{p}}_{\mathrm{target}}=\arg\max_{p\in \{p_{1},\ldots ,p_{T_{\max}}\}}L\left(p\right)$

### 4.4. Computational Complexity Analysis

In this section, we analyze the computational complexity of the proposed method during its operational online phase. The offline training phase, while computationally intensive, is executed only once to establish the model parameters. Therefore, our analysis focuses exclusively on the inference stage, as its efficiency is paramount for real-time applicability. The computational burden of the online phase is primarily determined by the forward propagation of an input sample through the network. Let us define the key variables that characterize the network’s architecture:

- $D_{in}$: the dimension of the input vector x.
- *L*: the total number of hidden layers in the network.
- $N_{l}$: the number of neurons in the *l*-th hidden layer, where l∈{1,2,…,L}.
- $D_{out}$: the dimension of the final output vector.

The online computation process for a single input sample $x\in R^{D_{in}}$ proceeds as follows: First, the computation between the input layer and the first hidden layer involves a matrix–vector multiplication with the weight matrix $W_{1}\in R^{N_{1}\times D_{in}}$ and the addition of a bias vector $b_{1}\in R^{N_{1}}$. The computational cost of this step is dominated by the matrix–vector product, which requires $O(D_{in}N_{1})$ floating-point operations (FLOPs). Next, for each subsequent hidden layer *l* (where *l* ranges from 2 to *L*), the computation involves transforming the activation output from the previous layer, $h_{l-1}\in R^{N_{l-1}}$, using the weight matrix $W_{l}\in R^{N_{l}\times N_{l-1}}$. The cost for each such layer is similarly dominated by the matrix–vector multiplication, amounting to $O(N_{l-1}N_{l})$ FLOPs. Finally, the output layer computes the final result by transforming the activations of the last hidden layer, $h_{L}\in R^{N_{L}}$, using the output weight matrix $W_{out}\in R^{D_{out}\times N_{L}}$. This step has a complexity of $O(N_{L}D_{out})$. The costs of bias additions and element-wise activation functions (e.g., ReLU or sigmoid) are $O(N_{l})$ and $O(D_{out})$, respectively. These are lower-order terms compared with the matrix–vector multiplications and can be omitted in the asymptotic analysis. By summing the costs of all layers, the total computational complexity for a single forward pass during the online phase is given by

(29) $$C_{online}=O\left(D_{in}N_{1}+\sum_{l=2}^{L}N_{l-1}N_{l}+N_{L}D_{out}\right)$$

This expression demonstrates that the online computational complexity is linear with respect to the number of parameters in the network. Such a linear relationship ensures that the model can perform inference efficiently, making it well-suited for applications requiring rapid response times, as the latency scales predictably with the model size. This complexity is well within the acceptable range for modern computational platforms.

### 4.5. Theoretical Guarantees

To theoretically evaluate the fundamental limit of positioning accuracy for the proposed system, we derive the Cramér–Rao Lower Bound (CRLB). The CRLB provides a lower bound on the variance of any unbiased estimator for a deterministic parameter, which in our case is the target’s position vector $p_{\mathrm{target}}\in R^{2}$. For an unbiased position estimator ${\hat{p}}_{\mathrm{target}}$, the error covariance matrix is bounded by the inverse of the Fisher Information Matrix (FIM), J:

(30) $$E\left[\left({\hat{p}}_{\mathrm{target}}-p_{\mathrm{target}}\right){\left({\hat{p}}_{\mathrm{target}}-p_{\mathrm{target}}\right)}^{⊤}\right]\geq J^{-1}$$

The trace of this matrix gives the lower bound on the mean squared error (MSE), which was briefly introduced in the original manuscript and is restated here for completeness:

(31) $$E[∥{\hat{p}}_{\mathrm{target}}-p_{\mathrm{target}}{∥}^{2}]\geq \mathrm{Tr}\left(J^{-1}\right)$$

The FIM, J, quantifies the amount of information that the observed feature vector $h_{\mathrm{target}}$ carries about the unknown position p. For a set of parameters $p={\left[p_{1},p_{2}\right]}^{⊤}$, the elements of the 2×2 FIM are given by the negative expectation of the second-order partial derivatives of the log-likelihood function with respect to those parameters:

(32) $$J_{ij}\left(p\right)=-E_{h_{\mathrm{target}}}\left[\frac{\partial^{2}\log p\left(h_{\mathrm{target}}|p\right)}{\partial p_{i}\partial p_{j}}\right]$$

In our framework, the likelihood $p(h_{\mathrm{target}}|p)$ is defined by the Gaussian Process posterior predictive distribution. Assuming independence across the *D* feature dimensions for analytical tractability, the total log likelihood is the sum of the individual log likelihoods from Equation (23):

(33) $$\begin{array}{cc} \log p(h_{\mathrm{target}}|p)\; & =\sum_{d=1}^{D}\log p\left(h_{\mathrm{target}}^{\left(d\right)}|p\right)\\ \; & =\sum_{d=1}^{D}\left[-\frac{{\left(h_{\mathrm{target}}^{\left(d\right)}-\mu^{\left(d\right)}\left(p\right)\right)}^{2}}{2\sigma_{d}^{2}\left(p\right)}-\frac{1}{2}\log\left(2\pi \sigma_{d}^{2}\left(p\right)\right)\right] \end{array}$$

where $\mu^{\left(d\right)}\left(p\right)$ and $\sigma_{d}^{2}\left(p\right)$ are the GP predictive mean and variance for the *d*-th feature dimension, as given in Equations (24) and (25). To compute the FIM, we take the partial derivatives of Equation (33) with respect to the position coordinates $p_{i}$ and $p_{j}$. Under the assumption that the estimator is evaluated at the true location $p_{\mathrm{target}}$, we have $E\left[h_{\mathrm{target}}^{\left(d\right)}\right]=\mu^{\left(d\right)}\left(p_{\mathrm{target}}\right)$. After performing the differentiation and taking the negative expectation, which simplifies the cross-terms involving $\left(h_{\mathrm{target}}^{\left(d\right)}-\mu^{\left(d\right)}\left(p\right)\right)$, the FIM elements are found to be

(34) $$J_{ij}\left(p\right)=\sum_{d=1}^{D}\left[\frac{1}{\sigma_{d}^{2}\left(p\right)}\frac{\partial \mu^{\left(d\right)}\left(p\right)}{\partial p_{i}}\frac{\partial \mu^{\left(d\right)}\left(p\right)}{\partial p_{j}}+\frac{1}{2\sigma_{d}^{4}\left(p\right)}\frac{\partial \sigma_{d}^{2}\left(p\right)}{\partial p_{i}}\frac{\partial \sigma_{d}^{2}\left(p\right)}{\partial p_{j}}\right]$$

This expression connects the theoretical performance limit directly to our model’s components. The terms $\frac{\partial \mu^{\left(d\right)}\left(p\right)}{\partial p_{i}}$ and $\frac{\partial \sigma_{d}^{2}\left(p\right)}{\partial p_{i}}$ are the gradients of the GP predictive mean and variance, which depend on the partial derivatives of the kernel function $K(p,p^{\prime })$ with respect to the coordinates of p. Therefore, the FIM is ultimately determined by the sensitivity of our hybrid covariance kernel to spatial changes. A higher gradient in the kernel function leads to a larger FIM and thus a lower CRLB, indicating higher potential positioning accuracy. In our experimental analysis, we compute this bound to benchmark the performance of the proposed estimator.

## 5. Experimental Validation

### 5.1. Experimental Setup and Data Acquisition

To validate the proposed positioning framework, we designed a comprehensive experimental protocol encompassing hardware configuration, data acquisition, and multi-scenario environmental validation. The entire setup was meticulously planned to ensure the collection of high-fidelity data and enable a rigorous evaluation of the algorithm’s performance under diverse and challenging conditions.

**Figure 2:**
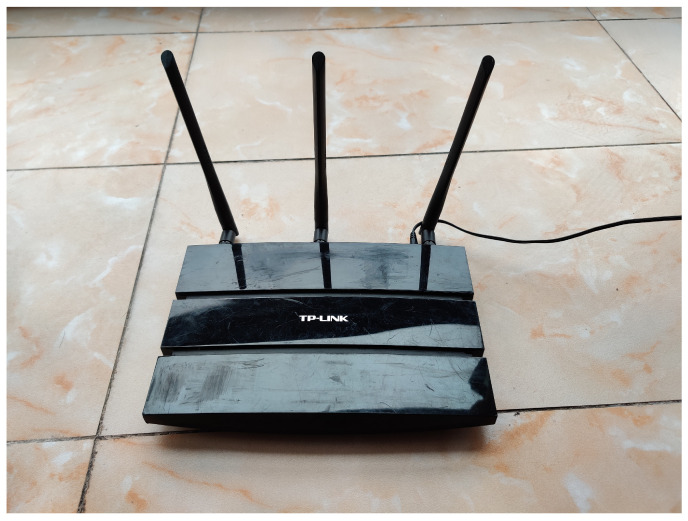
TP-Link WDR4310 router with OpenWrt firmware.

The acquisition of Channel State Information (CSI) was performed using a TP-Link WDR4310 router (TP-Link Technologies Co., Ltd., Shenzhen, China) flashed with the OpenWrt open-source firmware (v22.03.3), as illustrated in Figure 2. This platform facilitates the capture of CSI measurements across 56 orthogonal frequency-division multiplexing (OFDM) subcarriers. To align with common real-world deployment, we implemented a 3 × 1 multiple-input single-output (MISO) architecture where the transmitter utilizes three omnidirectional antennas and the receiver employs a single antenna.

**Figure 3:**
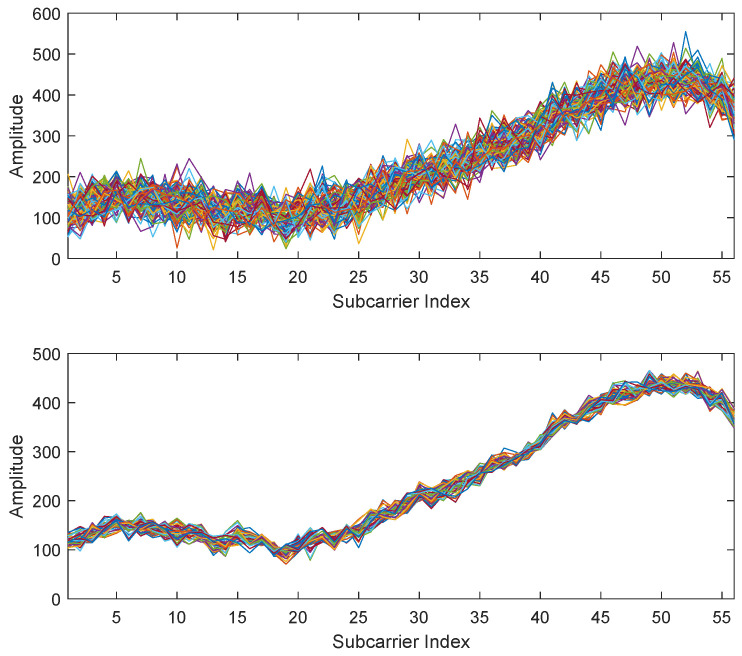
Signal preprocessing results: (**Top**) Raw CSI amplitude measurements showing multipath-induced fluctuations. (**Bottom**) Denoised signal after wavelet decomposition and skewness normalization.

The raw CSI measurements, inherently susceptible to significant non-Gaussian noise from multipath propagation and hardware non-linearities, were processed using the adaptive filtering pipeline detailed in Section 4.1.2. This crucial step validates the integrity of the fingerprint database prior to model training and feature extraction. As illustrated in Figure 3, the framework provides a quantifiable improvement in data quality: at a representative test location, it achieved an 8.78 dB reduction in noise variance—equivalent to an 86.8% reduction in noise power—while retaining 98.2% of the original signal energy. By significantly enhancing the signal-to-noise ratio (SNR) without losing the discriminative amplitude characteristics essential to positioning, this preprocessing step establishes the high-fidelity data foundation necessary for the robust performance of the subsequent modeling stages.

**Figure 4:**
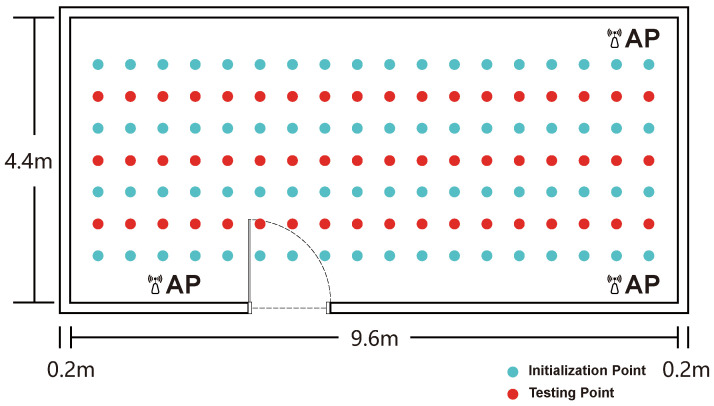
The experimental setup of the empty environment.

**Figure 5:**
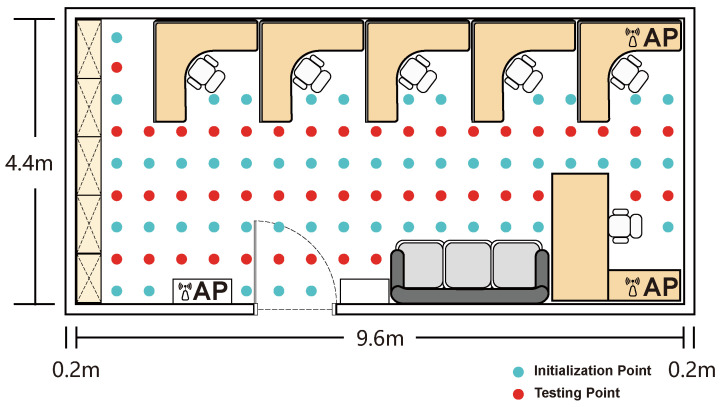
The experimental setup of the office environment.

**Figure 6:**
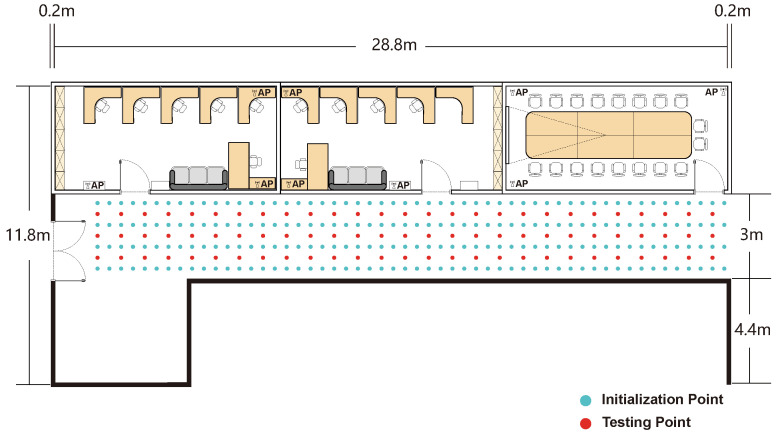
The experimental setup of the corridor environment.

To systematically evaluate the robustness and practical performance of our localization methodology, experimental validations were conducted in three distinct scenarios, specifically chosen to represent a spectrum of environmental complexity. The first, an empty chamber (9.6 × 4.4 m), served as a baseline to assess performance under near-ideal, Line-of-Sight (LOS) conditions, using a grid of 72 initialization and 54 test points (Figure 4). To introduce realistic challenges, subsequent evaluations were performed in an office space (9.6 × 4.4 m) with typical furniture and obstructions causing complex multipath propagation (50 initialization and 44 test points; Figure 5) and an adjacent corridor (28.8 × 3 m). The corridor, with its elongated geometry and potential for severe signal attenuation, represents a common yet difficult indoor navigation scenario, for which an expanded dataset of 216 initialization and 81 test points was collected (Figure 6). In all scenarios, the test locations were methodically selected to ensure comprehensive spatial coverage with a consistent 0.5-m separation between adjacent points.

**Figure 7:**
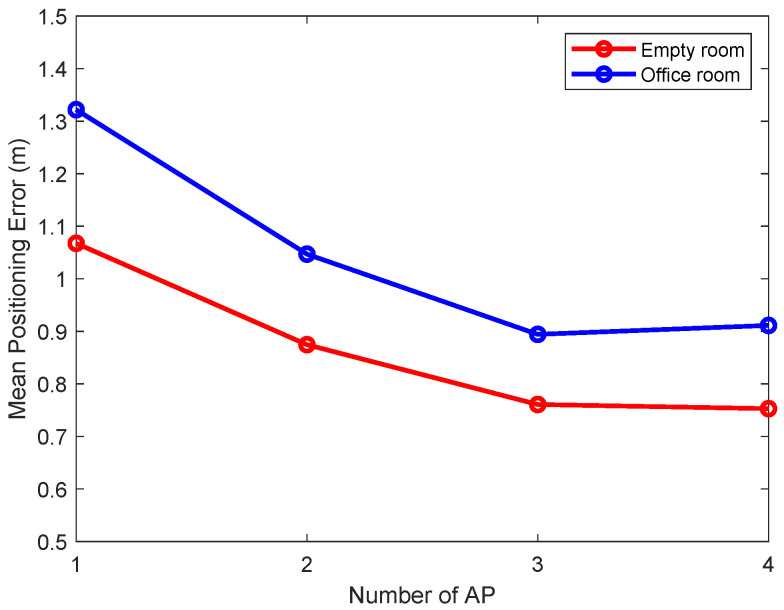
Variation in average positioning error with number of access points.

The number of deployed access points (APs) is a critical parameter that dictates the trade-off between positioning accuracy and practical deployment cost. To establish a methodologically sound configuration for our main experiments, a preliminary study was conducted to determine an optimal AP quantity. As shown in Figure 7, we evaluated the average localization error while varying the number of APs from one to four. The results clearly indicate that performance improves significantly up to three APs, after which the accuracy gains diminish. Based on this empirical evidence, a three-AP configuration—with APs strategically deployed as shown in Figure 4, Figure 5 and Figure 6—was adopted for all scenarios. This data-driven decision ensures that the subsequent comparative analysis is conducted under conditions representing an optimal balance between performance and complexity, thereby enabling a fair and robust evaluation against other methods.

### 5.2. Results and Discussion

**Figure 8:**
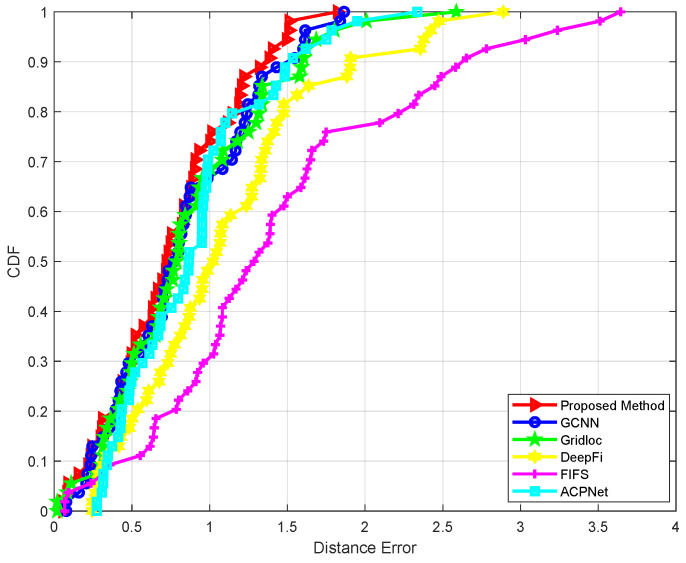
The CDF of positioning errors in the empty room.

**Figure 9:**
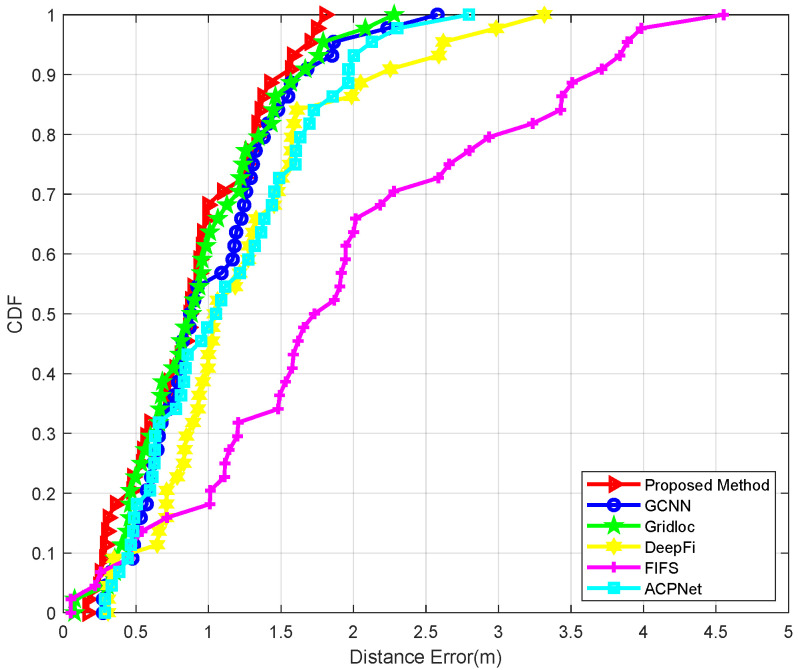
The CDF of positioning errors in the office room.

**Figure 10:**
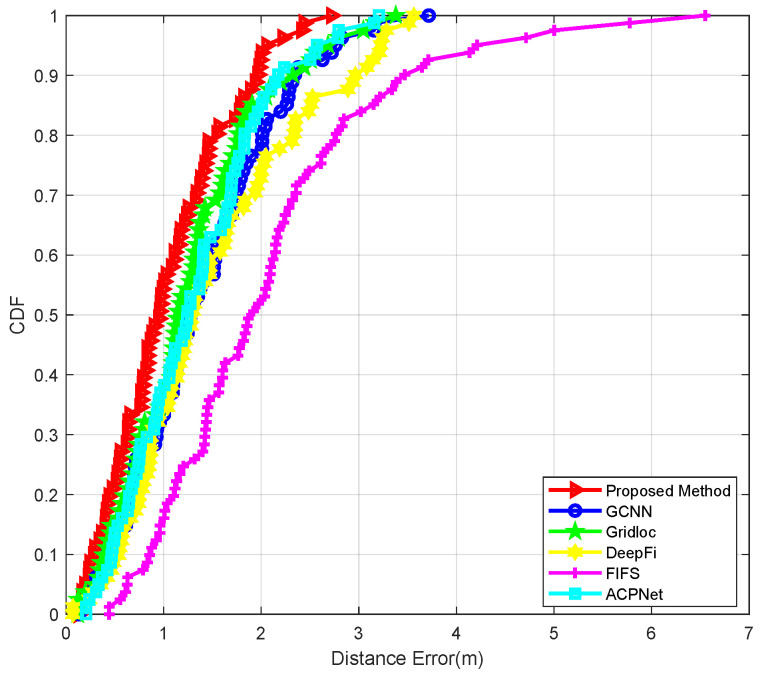
The CDF of positioning errors in the corridor environment.

**Figure 11:**
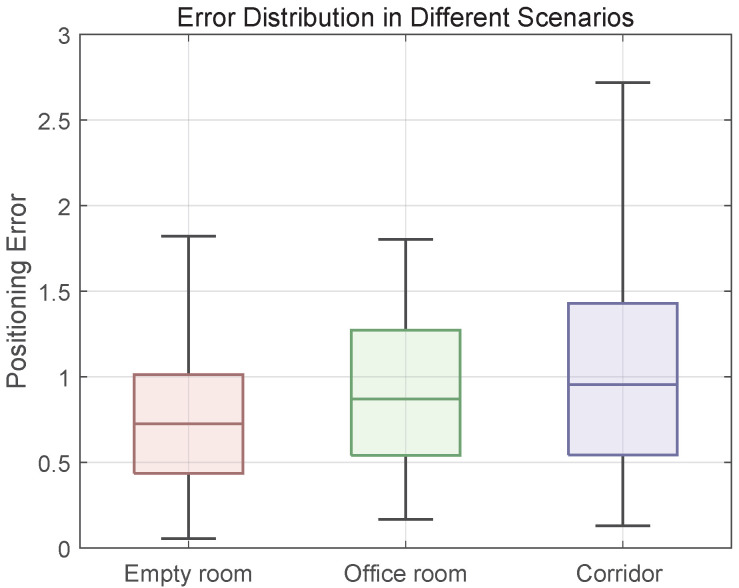
The error distribution of the proposed method in 3 different scenarios.

In addition to the methodology presented in this work, five state-of-the-art localization techniques were employed for comparative analysis in test point positioning: the conventional fingerprinting technique (FIFS) proposed in Reference [16], which utilizes traditional radio fingerprint matching; the Deep Neural Network-based approach (DeepFi) introduced in Reference [12], employing classical depth learning architectures for signal pattern recognition; the Attention-Aided Deep Learning network (ACPNet) from Reference [27], which leverages attention mechanisms to refine CSI features; the hybrid localization methodology (Gridloc) developed in Reference [15], which synergistically combines Received Signal Strength Indicator (RSSI) measurements with Channel State Information (CSI) data fusion; and the graph convolutional neural network architecture (GCNN) presented in Reference [31], implementing advanced graph-based learning for spatial feature extraction. Quantitative performance metrics, including mean positioning error, standard deviation of the error, and 90% accuracy thresholds (i.e., the error value below which 90% of test points fall), are summarized for each method across the different environments in Table 1, Table 2 and Table 3. The error distributions are further visualized through Cumulative Distribution Functions (CDFs) in Figure 8, Figure 9 and Figure 10. Figure 11 specifically illustrates the error distributions achieved by the method proposed in this paper across the three distinct scenarios. These results visually corroborate the expectation that an increase in environmental complexity, such as the presence of obstacles and the need for signal penetration through walls, generally leads to a reduction in the stability of received signals and, consequently, an escalation in positioning errors. This figure provides a crucial self-benchmark, illustrating how the proposed method’s performance intrinsically responds to increasing environmental challenges, which then serves as a basis for evaluating its relative robustness compared with other techniques.

In the empty room scenario (Table 1), which represents a near-ideal environment with minimal multipath interference, the proposed method achieved superior performance with a mean error of 0.7608 m. This result underscores the fundamental efficacy of our model’s feature extraction and spatial modeling capabilities even under baseline conditions. In contrast, the conventional FIFS method exhibited the highest mean error (1.4557 m), attributable to its reliance on averaged CSI amplitude features that fail to address temporal–spatial signal dynamics. DeepFi (1.1157 m) and the newly introduced ACPNet (0.9034 m) demonstrated the advantages of using deep learning for feature extraction. Notably, ACPNet’s use of intra-sample attention mechanisms to weigh subcarriers and antennas allows it to refine CSI features more effectively than DeepFi’s single-scale architecture, resulting in better accuracy. However, both GCNN (0.8350 m) and Gridloc (0.8746 m) outperformed ACPNet. This is because, as argued in our related work review, ACPNet’s “inward-facing” attention operates only within a single CSI sample and does not explicitly model the spatial relationships between different APs. Methods like GCNN, which model the physical topology, and the proposed method, which models the signal manifold, gain a distinct advantage by capturing this crucial inter-AP spatial information, leading to lower positioning errors even in this relatively simple environment.

In the office environment with obstacles (Table 2), the introduction of complex multipath propagation provides a more rigorous test of each model’s robustness. The proposed method maintained its leading performance with a mean error of 0.8943 m, showing a modest degradation of only 17.5% compared with the empty room. This resilience highlights the effectiveness of our framework’s skewness-adaptive filtering and dynamic Kalman updates in suppressing the non-Gaussian noise prevalent in such environments. The performance gap between our method and others widened significantly. For instance, ACPNet’s mean error increased to 1.1517 m, a performance degradation of 27.5%. This larger increase empirically validates the critique of its architecture: in an environment with obstructions, the reliability of signals from different APs varies drastically. ACPNet’s inability to model these dynamic inter-AP correlations means it cannot adaptively fuse features from the most reliable sources, leading to a substantial drop in accuracy. In contrast, our method’s graph attention fusion mechanism is explicitly designed to learn these correlations, providing superior robustness. While Gridloc’s hybrid approach (0.9538 m) offered some adaptability, its reliance on Euclidean metrics for spatial relationships still resulted in a 6.6% higher mean error than our geometrically aware model.

In the elongated corridor scenario (Table 3), where severe signal attenuation and geometrically constrained propagation paths challenge conventional models, the proposed method’s core innovation demonstrates its full value. It achieved a mean error of 1.0433 m, outperforming the next-best method, Gridloc (1.2716 m), by 21.9%. The performance of Euclidean-based models degraded significantly in this non-Euclidean environment. Notably, GCNN (1.4009 m) performed worse than ACPNet (1.3235 m). This reversal is highly instructive: GCNN’s graph model, which assumes that Euclidean distance is a meaningful proxy for signal similarity, is fundamentally misled by the corridor’s geometry. The actual signal propagation paths are constrained by the walls, creating a hierarchical structure that flat space cannot represent. In this extreme case, GCNN’s flawed spatial model becomes more detrimental than ACPNet’s approach of simply refining features without an explicit inter-AP model. This result provides a powerful justification for our method’s central contribution: the hyperbolic covariance kernel. By representing fingerprints in a negatively curved space that naturally accommodates hierarchical, tree-like structures, our framework preserves the intrinsic manifold of signal propagation. This geometric fidelity prevents the critical information loss suffered by all other methods, enabling superior accuracy in the most challenging indoor environments.

To further assess the statistical efficiency of the proposed method, we compare its empirical performance against the theoretical Cramér–Rao Lower Bound (CRLB) derived in Section 4.5. The CRLB represents the minimum achievable mean squared error for any unbiased estimator and thus serves as an ideal performance benchmark. We calculated the FIM (Equation (34)) and the corresponding CRLB (Equation (31)) at the true location of each test point in our three experimental environments. Table 4 summarizes the average CRLB for each scenario and compares it with the mean positioning error of our method.

As shown in Table 4, our method achieves a mean error that is reasonably close to the CRLB, with the gap increasing from 0.11 m in the empty room to 0.19 m in the corridor. This trend is expected, as unmodeled environmental complexities introduce biases that prevent any real-world estimator from perfectly attaining the bound. In all scenarios, the mean error of our method is close to the CRLB, indicating high efficiency for a majority of the test points. The divergence in the tail of the distribution reflects the challenging NLOS conditions for a subset of points, where the GP model’s assumptions are most stressed. Overall, this analysis confirms that the proposed estimator operates near the fundamental performance limit dictated by the information content of the CSI signals.

## 6. Conclusions

This paper presents a novel Wi-Fi indoor positioning framework that significantly enhances accuracy and robustness by addressing key limitations in existing methods. The synergy of three innovations is central to our approach: (1) a skewness-adaptive filtering pipeline for effective non-Gaussian noise suppression; (2) a graph attention network for correlation-aware feature fusion from multiple APs; and (3) a hyperbolic covariance model that preserves the intrinsic manifold structure of signal propagation in complex environments. Experimental results validate the superiority of our method, especially in obstructed scenarios where the hyperbolic spatial representation demonstrates exceptional resilience. However, we acknowledge the limitations of this study. Our validation was conducted using a homogeneous hardware setup within single-floor environments, thus the generalizability to large-scale, multi-floor venues and across heterogeneous devices warrants further investigation. Future work will focus on extending the hyperbolic model to 3D space for multi-floor positioning and exploring domain adaptation techniques to create a hardware-agnostic framework. Furthermore, integrating our model with semi-supervised learning and edge computing will be explored to reduce recalibration efforts in dynamic environments and enhance real-time deployment feasibility.

## Figures and Tables

**Table 1:** The mean positioning errors and corresponding standard deviations across different positioning methods (empty room).

Positioning Method	Error Metrics		
	Mean Error (m)	Standard Deviation (m)	90% Acc. (m)
Proposed Method	0.7608	0.1761	1.3844
DeepFi	1.1157	0.3933	1.9518
FIFS	1.4557	0.7290	2.6652
GCNN	0.8350	0.2140	1.5532
Gridloc	0.8746	0.2801	1.6064
ACPNet	0.9034	0.2179	1.5446

**Table 2:** The mean positioning errors and corresponding standard deviations across different positioning methods (office room).

Positioning Method	Error Metrics		
	Mean Error (m)	Standard Deviation (m)	90% Acc. (m)
Proposed Method	0.8943	0.2026	1.5667
DeepFi	1.2645	0.4725	2.2875
FIFS	1.9501	1.3035	3.7244
GCNN	1.0323	0.2667	1.6978
Gridloc	0.9538	0.2514	1.6767
ACPNet	1.1517	0.3673	1.9691

**Table 3:** The mean positioning errors and corresponding standard deviations across different positioning methods (corridor environment).

Positioning Method	Error Metrics		
	Mean Error (m)	Standard Deviation (m)	90% Acc. (m)
Proposed Method	1.0433	0.3800	1.9677
DeepFi	1.5229	0.7823	3.0115
FIFS	2.0945	1.3737	3.5409
GCNN	1.4009	0.5942	2.3599
Gridloc	1.2716	0.5352	2.3692
ACPNet	1.3235	0.4910	2.1994

**Table 4:** Comparison of the proposed method’s mean positioning error with the theoretical CRLB.

Environment	Proposed Method’s Mean Error (m)	Average CRLB (m)
Empty Room	0.7608	0.6512
Office Room	0.8943	0.7325
Corridor	1.0433	0.8561

## Data Availability

The data supporting the reported results in this study are not publicly available due to privacy and ethical restrictions. However, additional details and datasets may be made available upon reasonable request to the corresponding author.

## Abbreviations

The following abbreviations are used in this manuscript:

ACPNet	Attention-Aided Deep Learning Network
AMT	Adapted Mean Teacher
AOA	Angle of Arrival
AP	access point
BO	Bayesian Optimization
CDF	Cumulative Distribution Function
CFR	Channel Frequency Response
CRLB	Cramér–Rao Lower Bound
CSI	Channel State Information
DeepFi	Deep Neural Network-based Fingerprinting
DNN	Deep Neural Network
EI	Expected Improvement
FIFS	Fine-Grained Indoor Fingerprinting System
FIM	Fisher Information Matrix
FLOPs	floating-point operations
GAT	graph attention network
GCNN	graph convolutional neural network
GNNs	Graph Neural Networks
GNSS	Global Navigation Satellite System
GP	Gaussian Process
Gridloc	hybrid localization methodology
IQR	Interquartile Range
LBS	location-based service
LOS	Line-of-Sight
MIMO	multiple-input multiple-output
MISO	multiple-input single-output
MSE	mean squared error
NLOS	Non-Line-of-Sight
OFDM	orthogonal frequency-division multiplexing
PDF	Probability Density Function
RFID	Radio-Frequency Identification
RSSI	Received Signal Strength Indicator
SNR	signal-to-noise ratio
TCN	Temporal Convolutional Network
TOF	Time of Flight
UWB	Ultra-Wideband
